# Edge-intelligent bimodal iontronic skin for human−robot collaboration

**DOI:** 10.1093/nsr/nwag194

**Published:** 2026-03-30

**Authors:** Daewon Yoon, Dae-Hyeong Kim

**Affiliations:** Center for Nanoparticle Research, Institute for Basic Science (IBS), Republic of Korea; School of Chemical and Biological Engineering, Institute of Chemical Processes, Seoul National University, Republic of Korea; Center for Nanoparticle Research, Institute for Basic Science (IBS), Republic of Korea; School of Chemical and Biological Engineering, Institute of Chemical Processes, Seoul National University, Republic of Korea

Human–machine interaction requires sensing systems that can detect physical stimuli and translate them into actionable signals for real-time control and decision-making [1,2]. Recent advances in soft bioelectronics have enabled flexible, skin-like platforms with multimodal sensing capabilities [3,4]. However, as these systems scale in spatial coverage and channel density, challenges such as signal crosstalk, slow readout and difficulty in real-time interpretation persist [5,6]. In particular, distinguishing human touch from object contact and enabling low-latency, on-device inference remain key barriers to practical human–robot collaboration. In a recent study, Prof. Chuanfei Guo and colleagues addressed these challenges with a bimodal iontronic skin integrated with edge intelligence, enabling real-time perception and intention-aware human–robot interaction [7].

**Figure 1.**
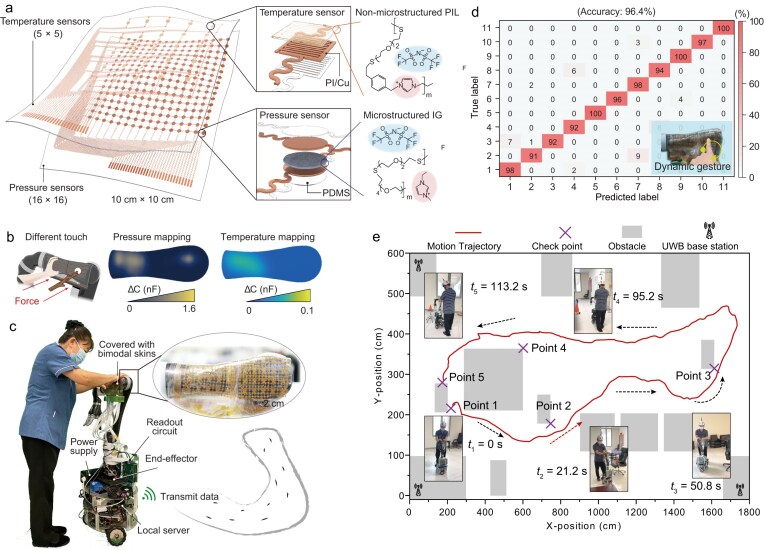
(a) Schematic illustration of the bimodal iontronic skin architecture. (b) Discrimination of human touch and object contact enabled by bimodal sensing. (c) Integration of a robot with large-area bimodal skins. (d) High-accuracy gesture recognition based on bimodal tactile signals. (e) Assisted walking enabled by the integrated bimodal sensing and edge intelligence. Reproduced from ref. [7] with permission.

At the device level, the system combines two complementary iontronic sensors within a stacked skin module: a microstructured ionogel-based pressure sensor and a polyionic-liquid temperature sensor (Fig. 1a). This architecture is significant because it does more than add an extra sensing channel. Pressure is sensed through capacitance changes arising from deformation-induced variations in contact area and effective dielectric properties in microstructured ionogel, enabling sensitive responses to changes in contact state. In contrast, temperature is sensed by a planar polyionic liquid layer whose response is largely insensitive to pressure and primarily reflects thermal stimuli. By orthogonally combining mechanical and thermal cues, the skin can distinguish a human hand from inanimate object contact, an ability that is particularly valuable for collaborative robots operating in shared environments (Fig. 1b). In this way, the bimodal design moves robotic skin beyond simple touch detection toward more context-aware interaction.

The conceptual advance becomes clearer at the system level. The authors mounted multiple skin modules onto a collaborative robotic arm, achieving a large-area coverage with 768 pressure-sensing units and 75 temperature-sensing units (Fig. 1c). To handle the high-dimensional spatiotemporal data streams generated by the dense sensor network, the system integrated a frequency-encoded multiplexed readout strategy with lightweight on-edge learning algorithms. This combination enabled the robot to acquire high-throughput spatiotemporal tactile data and to process and interpret it locally without reliance on external computation for motion-intention estimation and contact-gesture recognition. Notably, the gesture-classification framework identified 11 interaction gestures with an average accuracy of 96.4%, demonstrating the viability of low-latency, on-device tactile intelligence on the robotic platform (Fig. 1d).

The practical value of this platform was illustrated in a human–robot assistive walking scenario, where the skin-covered robot arm supported physical collaboration with a user during walking. Importantly, the system leveraged bimodal sensing for motion guidance, intention inference and safety-related responses. Temperature readout served as a safeguard to verify that the contact originated from a human hand, while gesture recognition enabled the system to detect a firm grip and trigger emergency braking when needed. In a path-following task involving a mobility-impaired participant, the robot responded to tactile interaction to assist navigation along a user-defined path (Fig. 1e). These demonstrations highlight how multimodal tactile sensing and edge inference can together support intuitive, safe, and reliable human–robot collaboration.

Overall, Guo and co-workers moved beyond simply increasing sensor density and instead offered a framework that links multimodal tactile transduction, scalable readout and edge-based intelligence within a single collaborative robotic system. By demonstrating that large-area pressure–temperature sensing can be directly mapped to real-time intention inference and assistive actuation, this study took a step toward practical deployment. Looking ahead, further advances in spatial resolution, multimodal integration and generalized learning across users and tasks will be essential to extend this strategy from arm-mounted interfaces to more fully embodied robotic platforms.
